# *Salmonella* Typhimurium and Multidirectional Communication in the Gut

**DOI:** 10.3389/fmicb.2016.01827

**Published:** 2016-11-22

**Authors:** Elena V. Gart, Jan S. Suchodolski, Thomas H. Welsh, Robert C. Alaniz, Ronald D. Randel, Sara D. Lawhon

**Affiliations:** ^1^Department of Veterinary Pathobiology, College of Veterinary Medicine, Texas A&M University, College StationTX, USA; ^2^Department of Small Animal Clinical Sciences, College of Veterinary Medicine, Texas A&M University, College StationTX, USA; ^3^Department of Animal Science, College of Agriculture and Life Sciences, Texas A&M University, College StationTX, USA; ^4^Department of Microbial Pathogenesis and Immunology, College of Medicine, Texas A&M Health Science Center, Texas A&M University, College StationTX, USA; ^5^Texas A&M AgriLife Research, OvertonTX, USA

**Keywords:** *Salmonella* Typhimurium, microbiota, quorum sensing, autoinducer-2, autoinducer-3, catecholamines, stress

## Abstract

The mammalian digestive tract is home to trillions of microbes, including bacteria, archaea, protozoa, fungi, and viruses. In monogastric mammals the stomach and small intestine harbor diverse bacterial populations but are typically less populated than the colon. The gut bacterial community (microbiota hereafter) varies widely among different host species and individuals within a species. It is influenced by season of the year, age of the host, stress and disease. Ideally, the host and microbiota benefit each other. The host provides nutrients to the microbiota and the microbiota assists the host with digestion and nutrient metabolism. The resident microbiota competes with pathogens for space and nutrients and, through this competition, protects the host in a phenomenon called colonization resistance. The microbiota participates in development of the host immune system, particularly regulation of autoimmunity and mucosal immune response. The microbiota also shapes gut–brain communication and host responses to stress; and, indeed, the microbiota is a newly recognized endocrine organ within mammalian hosts. *Salmonella enterica* serovar Typhimurium (*S.* Typhimurium hereafter) is a food-borne pathogen which adapts to and alters the gastrointestinal (GI) environment. In the GI tract, *S.* Typhimurium competes with the microbiota for nutrients and overcomes colonization resistance to establish infection. To do this, *S.* Typhimurium uses multiple defense mechanisms to resist environmental stressors, like the acidic pH of the stomach, and virulence mechanisms which allow it to invade the intestinal epithelium and disseminate throughout the host. To coordinate gene expression and disrupt signaling within the microbiota and between host and microbiota, *S.* Typhimurium employs its own chemical signaling and may regulate host hormone metabolism. This review will discuss the multidirectional interaction between *S.* Typhimurium, host and microbiota as well as mechanisms that allow *S.* Typhimurium to succeed in the gut.

## Introduction to *S.* Typhimurium Pathogenesis and Virulence

**FIGURE 1:**
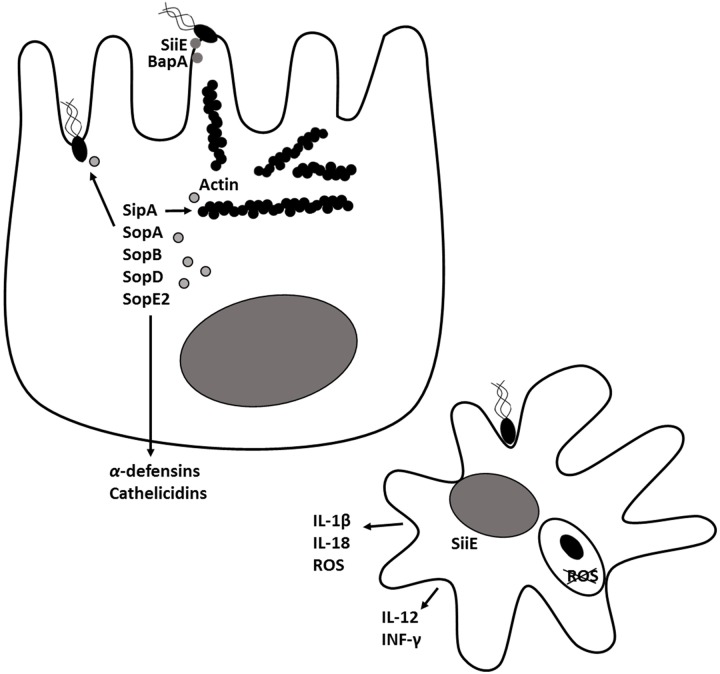
***Salmonella* Typhimurium pathogenesis and virulence.** In the intestinal lumen, *Salmonella* uses flagella to move close to the intestinal epithelial cells and uses fimbriae and adhesins (SiiE, BapA) for intimate cell attachment. Through, the type III secretion system encoded on pathogenicity island 1 (T3SS-1), *Salmonella* injects effector proteins SipA, SopA, SopB (SigD), SopD and SopE2 into host cells where they trigger cytoskeletal rearrangement, bacterial engulfment and formation of *Salmonella*-containing vacuole (SCV). The T3SS-1 effectors induce secretion of inflammatory cytokines and antimicrobial peptides by epithelial cells. A second type III secretion system encoded on *Salmonella* pathogenicity island 2 (T3SS-2) is expressed within the SCV. Proteins secreted through T3SS-2 prevent production of reactive oxygen species (ROS) and enables *Salmonella* to survive inside macrophages.

Salmonellosis in humans and food animals caused by *S.* Typhimurium is characterized by fever, acute intestinal inflammation, and diarrhea within 24 h after infection. *Salmonella* employs multiple virulence factors to overcome colonization resistance and induce intestinal inflammation (Fabrega and Vila, 2013). After entering the intestinal lumen, *Salmonella* uses flagella to move to the proximity of the intestinal epithelial cells and uses fimbriae for intimate cell attachment (**Figure 1**). Fimbriae bind the extracellular matrix glycoprotein laminin and mediate adhesion to the host cell. The autotransporter protein, MisL (Kingsley et al., 2000; Dorsey et al., 2005), binds to fibronectin and *Salmonella* adhesins (SiiE and BapA) allow the bacteria to tightly adhere to the intestinal epithelium (Fabrega and Vila, 2013). *Salmonella* pathogenicity islands 1 (SPI-1) and 2 (SPI-2) encode two type III secretory systems (T3SS) that are syringe-like apparatuses *Salmonella* uses to translocate bacterial proteins into host cells. The SPI-1 T3SS (T3SS-1) is associated with invasion of epithelial cells. Structural proteins build the molecular syringe structure of the T3SS. *Salmonella* injects effector proteins SipA, SopA, SopB (SigD), SopD, and SopE2 via the needle into the host cell where they trigger cytoskeletal rearrangement and bacterial engulfment (reviewed in detail by Notti and Stebbins, 2016). The T3SS-1 effectors also induce fluid secretion and promote inflammation (Thiennimitr et al., 2012). Throughout the invasion process, signaling via pathogen-associated molecular patterns such as flagella and lipopolysaccharide (LPS) induces inflammation. Once inside *Salmonella*-containing vacuoles (SCVs), *Salmonella* induces expression of a second T3SS, encoded on SPI-2. In epithelial cells, *Salmonella* can persist within or escape from the SCV to replicate in the cytoplasm. In macrophages, which are naturally phagocytic, *Salmonella* interferes with the assembly of the NADPH oxidase complex in the phagosomal membrane, thereby preventing superoxide production and allowing the bacteria to survive inside the cell (Barrow and Methner, 2013). Concomitant with invasion, epithelial cells, mononuclear cells and complement recognize *Salmonella* and other pathogens and trigger IL-1β, IL-12, IL-18, IL-23, TNF-α, INF-γ, and C5a production. These signals instruct the host to implement antibacterial responses including macrophage activation, recruitment of neutrophils, and release of antimicrobial peptides such as α-defensins and cathelicidins by epithelial cells. Activated macrophages and neutrophils release reactive oxygen radicals that are toxic to commensal microbiota but *S*. Typhimurium detoxifies (Bang et al., 2005; Crouch et al., 2005; Richardson et al., 2009; Winter et al., 2010). Therefore, in a hostile take-over, *S*. Typhimurium induces an inflammatory immune response which not only creates new resources like tetrathionate for *S.* Typhimurium, but also reduces resident microbiota thereby making already existing resources available for *S.* Typhimurium. Hence, the *Salmonella* induces an inflammatory immune response that allows it to compete with commensal microbiota and effectively colonize the gut (Hallstrom and McCormick, 2011; Fabrega and Vila, 2013).

## Multidirectional Signaling in the Gut and Effect on *S.* Typhimurium

Quorum sensing (QS) is a method of bacterial cell-to-cell signaling which allows the bacteria to coordinate gene expression at the population level. Receptors on bacterial cells recognize secreted molecules and initiate expression of downstream genes, including those involved in synthesis of QS compounds.

In the gastrointestinal environment bacteria produce hormones and hormone-like substances (Roshchina, 2010; Lyte, 2014) or modify the host’s signaling molecules, thus, affecting the host (Asano et al., 2012; Yano et al., 2015). Additionally, interspecies and interkingdom signaling modulate bacterial growth and virulence of pathogenic bacteria (Lee and Lee, 2010; Sandrini et al., 2015).

### *Salmonella* Typhimurium Autoinducers and their Role in Virulence

#### Autoinducer-2

More than a decade ago Surette et al. (1999), discovered that *Escherichia coli* and *S.* Typhimurium secrete a small, soluble, heat-labile, signaling molecule, which was named autoinducer-2 (AI-2). The signal concentration in growth media was maximal at the mid-exponential phase yet disappeared from the media at the beginning of the stationary phase in coordination with glucose depletion (Surette and Bassler, 1998). Additionally, AI-2-dependent signaling required low pH and high osmolality, whereas low osmolality induced signal degradation (Surette and Bassler, 1999).

**FIGURE 2:**
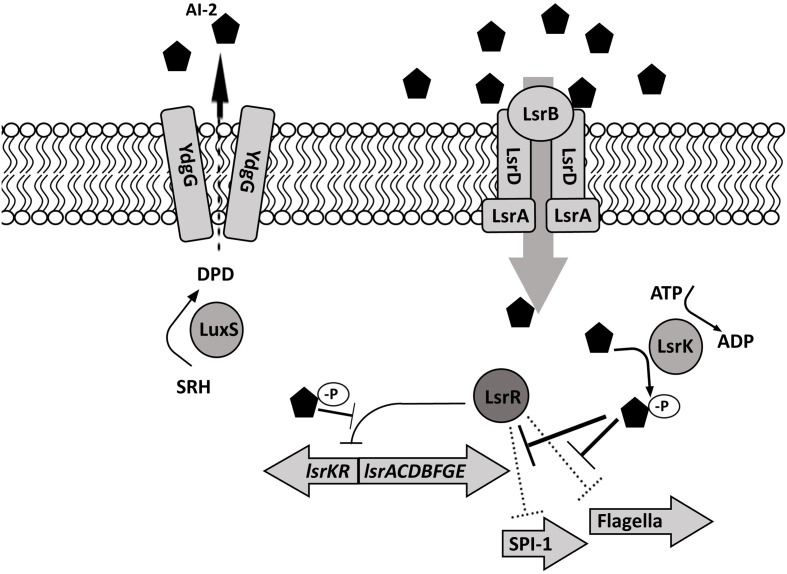
***Salmonella* Typhimurium autoinducer-2 (AI-2) signaling.** The signaling molecule AI-2 is produced by LuxS during an exponential growth, is exported by an unknown mechanism (possibly by YdgG), and accumulates extracellularly. Extracellular AI-2 is sensed by the *lsr*-encoded transport cassette and is actively transported into the bacterial cell. Following intracellular phosphorylation by LsrK, AI-2 negatively regulates the transcriptional repressor protein LsrR, allowing the transcription of *lsr*- and SPI-1 – encoded genes as well as flagellar genes.

Autoinducer-2 production in *S.* Typhimurium depends on a series of enzymatic reactions (**Figure 2**). First, methyl transferases convert *S*-adenosyl methionine (SAM) to *S*-adenosylhomocysteine (SAH), then methylthioadenosine/*S*-adenosylhomocysteine nucleosidase (Pfs) and *S*-ribosylhomocysteinase (LuxS) convert SAH to 4,5 dihydroxy-2,3-pentanedione (DPD). Finally, DPD is cyclized into (2R, 4S)-2-methyl-2,3,3,4-tetrahydroxytetra-hydrofuran (R-THMF, AI-2) (Taga et al., 2003; Miller et al., 2004). *Salmonella* Typhimurium produces and releases the majority of the AI-2 during exponential growth (Taga et al., 2003) and membrane transport protein YdgG may be involved in the extracellular transport of AI-2 (Herzberg et al., 2006). Extracellular AI-2 binds to autoinducer binding protein LsrB and is transported into bacterial cell via the ATP transporter encoded on *lsr* operon by *lsrACDB* (Xue et al., 2009). The phosphoenol pyruvate phosphotransferase system (PTS) is essential for initial ABC transporter activation and AI-2 internalization (Pereira et al., 2012). A cytoplasmic kinase, LsrK, phosphorylates internalized AI-2 (Taga et al., 2003). Phosphorylated AI-2 inactivates the transcriptional repressor protein LsrR in a dose-dependent manner (Xue et al., 2009) and induces *lsrACDBFGE* operon transcription (Taga et al., 2003). The LsrR protein, encoded on the *lsr* operon, binds two loosely conserved sites on *lsr*, thus, repressing the transcription of *lsrACDBFGE* and itself (Xue et al., 2009). Phospho-AI-2 is degraded by LsrG and LsrF. Since the *lsr* expression is delayed but not completely halted in a *lsrB* mutant, there may be an alternative AI-2 transporter in *S*. Typhimurium (Taga et al., 2003). Transcriptome analysis suggests that *rbsB* gene encoding ABC superfamily D-ribose transport protein may be involved in the AI-2 transport in *S.* Typhimurium (Jesudhasan et al., 2010). In *S.* Typhimurium, LsrR, also negatively regulates expression of genes involved in the oxidative stress response (*sodA, sodCI, sodCII*), which, in turn lowers the bacterial ability to survive within macrophages (Choi et al., 2010). Additionally, LsrR represses flagella expression and invasion. Inactivation of LsrR by the presence of phosphorylated AI-2 allows SPI-1 (*invF, sicA, sopB, sopE*) and flagella (*fliC, fliD*) gene transcription (Choi et al., 2012).

Although, the *lsr* operon is the only system known to be directly regulated by AI-2 in *E. coli* and *Salmonella*, proteomic analysis revealed that deletion of *luxS* in EHEC affected a variety of cell functions, including cell signaling, metabolism, information storage and processing, possibly through repressed tryptophan biosynthesis (Soni et al., 2007). More than 500 genes were differentially expressed between wild type (WT) *S.* Typhimurium and a *luxS* mutant *in vitro* (Jesudhasan et al., 2010). Deletion of *luxS* decreased the expression of genes encoding flagellar motility and chemotaxis as well as genes encoded on SPI-1 and *lsr*; however, the expression of *hilD*, *hilA*, *sipB*, and *invABCE* virulence factors increased (Jesudhasan et al., 2010). Phenotypically, deletion of *luxS* resulted in a decreased motility and virulence *in vitro*, which was restored by AI-2 supplementation in the media (Choi et al., 2007). A *S*. Typhimurium *luxS* mutant was also defective for epithelial cell invasion and virulence in mice (Choi et al., 2007). A combination of signals can modify AI-2 dependent virulence in *S.* Typhimurium. For example, *luxS* mutants have been shown to grow poorly in nutrient-poor M9 minimal medium. The addition of AI-2 restored growth of *luxS* mutants in minimal media but did not restore their ability to invade or survive in macrophages. Supplementation of the minimal media with both AI-2 and with long chain fatty acids (linoleic, oleic, palmitic, stearic) restored growth and macrophage invasion (Widmer et al., 2012). This connection between AI-2 and nutrient utilization suggests that, in addition to QS molecules, nutrient availability under complex conditions such as those encountered in the gut may affect *S.* Typhimurium signaling and virulence.

Interestingly, *E. coli* is able to intercept extracellular AI-2 produced by other bacterial species *in vitro* (Peixoto et al., 2014). As the microbiota produces AI-2 like molecules (Mitsumori et al., 2003), it is possible that *Enterobacteriaceae* can utilize interspecies signaling in the gut. In *E. coli*, chemotaxis to AI-2 depends on the L-serine receptor, Tsr, and the AI-2-binding protein, LsrB (Hegde et al., 2011). In addition to regulation of virulence, increased concentrations of AI-2 promoted plasmid exchange between two different strains of *E. coli* (Cho et al., 2003) suggesting that AI-2-dependent QS may facilitate genetic information exchange, although this has not been directly shown in *S.* Typhimurium.

#### AI-3/NE/Epi

**FIGURE 3:**
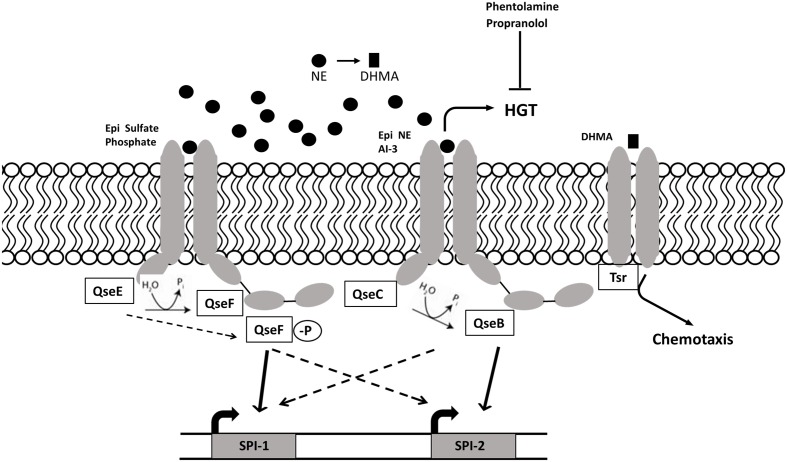
**AI-3/NE/Epi quorum sensing in *S*. Typhimurium.** Intestinal catecholamines are sensed by *S.* Typhimurium QseC/B and QseE/F regulatory systems. The sensor kinase QseE detects Epi, which leads to phosphorylation of response regulator QseF and subsequent induction of cell invasion genes encoded on SPI-1. Sensor kinase QseC autophosphorylates upon sensing Epi, NE and AI-3, and activates genes encoded by SPI-2 required for intracellular survival) through dephosphorylated response regulator QseB. QseE and QseC indirectly regulate SPI-2 and SPI-1 through QseB and QseF respectively. QseC-dependent increase in horizontal gene transfer and motility can be blocked by adrenergic antagonists phentolamine and propranolol. NE metabolite DHMA is sensed by serine chemoreceptor Tsr and induces chemotaxis.

Sperandio et al. (2003) discovered autoinducer 3 (AI-3) production by *Enterobacteriaceae*; however, the synthetic pathway and chemical formula of AI-3 are still unknown (Walters et al., 2006). The two-component regulators associated with AI-3 are also associated with recognition of host catecholamines (CAs) epinephrine (Epi), and norepinephrine (NE). *Salmonella* and *E. coli* respond to host catecholamines through two-component regulatory systems. *Salmonella* Typhimurium encodes orthologs of the *E. coli* two-component regulatory systems QseC/B (PreB/PreA) and QseF/E (Merighi et al., 2006; Pullinger et al., 2010a), where QseC and QseE are histidine sensor kinases and QseB and QseF are, respectively, their associated response regulators. QseC is able to sense AI-3 as well as Epi and NE, while QseE can recognize Epi and NE along with sulfate and phosphate (Moreira and Sperandio, 2012). The adrenergic histidine kinase, QseC, autophosphorylates upon binding to AI-3/Epi/NE and then dephosphorylates the response regulator QseB, inducing SPI-2 gene expression (Bearson et al., 2010; Moreira et al., 2010). The QseC/QseB two-component regulatory system controls virulence factors such as motility, invasion through SPI-1 genes and survival in macrophages through SPI-2, genes (Moreira et al., 2010). QseE senses NE, Epi, phosphate, and sulfate and acts on the response regulator QseF, which induces the expression of SPI-1 genes (**Figure 3**). The Sperandio group proposed a QseC/QseE interplay model, where QseC phosphorylates QseF, thus indirectly controlling motility and invasion virulence factors encoded by SPI-1. Overall, it seems that QseE plays an important role during epithelial cell invasion, while QseC is more important in systemic disease and intramacrophage replication (Moreira and Sperandio, 2012). The QseC-dependent signaling can be blocked by the α-adrenergic antagonist phentolamine (Clarke et al., 2006); however, the effect of adrenergic antagonists on QseE is yet to be determined.

In *Salmonella*, catecholamines induce growth in iron-restricted media (Freestone et al., 2007; Pullinger et al., 2010a) and facilitate the expression of genes encoded on SPI-1 (*sipA, sipB*) and SPI-3 (*mgt*) thus modulating virulence of *S.* Typhimurium *in vitro* and *in vivo* (Moreira et al., 2010). It is unclear whether NE-dependent enhancement of bacterial growth, motility and virulence depends on QseC/E signaling (Moreira et al., 2010; Pullinger et al., 2010a). It has been shown that the alpha-adrenergic antagonist, phentolamine, and the beta-adrenergic antagonist, labetalol, can neutralize norepinephrine-induced enhancement of motility (Bearson et al., 2010; Yang et al., 2014) suggesting similarity between bacterial and mammalian targets for adrenergic receptors. It is important to note that the chemoattractant properties of NE are in fact attributed to a bacterial NE metabolite, 3,4-dihydrohymandelic acid (DHMA). When the bacterial cell senses NE via QseC, the cell induces transcription of tyramine oxidase (*tynA*) and aromatic aldehyde dehydrogenase (*feaB*), which are involved in 3,4-dihydroxyphenylglycolaldehyde (DOPEGAL) and DHMA production. The latter is sensed by the bacterial serine chemoreceptor Tsr, which induces concentration-dependent chemotaxis of *E. coli* (Pasupuleti et al., 2014). Catecholamines may also play a role in transfer of antimicrobial resistance between *Salmonella* and *E. coli*, as physiological concentrations of NE (5 μM) have been shown to enhance plasmid transfer between *S.* Typhimurium and commensal *E. coli* in iron-rich lysogeny broth (LB) broth. Phentolamine, an α-adrenergic antagonist, and propranolol, a beta-adrenergic antagonist, inhibited NE-induced plasmid transfer (Peterson et al., 2011). Finally, catecholamine signaling may be involved in AI-2-dependent virulence regulation in *Enterobacteriaceae* as microarray analysis revealed that NE and Epi decrease the expression of *lsr* operon in *E. coli* O157:H7 *in vitro* (Bansal et al., 2007).

### Interspecies and Interkingdom Communication

#### Acyl-Homoserine Lactone

Several bacterial species such as *Vibrio cholera*, *Klebsiella pneumoniae*, and *Pseudomonas aeruginosa* utilize acyl-homoserine lactone (AHL) signaling (Frederix and Downie, 2011). In gram negative bacteria AHL is synthesized by LuxI, which binds to its cognate receptor LuxR, which subsequently binds specific DNA promoters and activates transcription of target genes (Federle and Bassler, 2003). Unexpectedly, in the mammalian gut, AHL was detected only in the bovine rumen where its concentration was correlated with the summer and spring seasons and colonization with *E. coli* O157:H7; however, it is not known which members of the rumen microbiota produce AHL (Edrington et al., 2009; Hughes et al., 2010).

In *S.* Typhimurium, AHLs stimulate biofilm formation by regulating glycogen biosynthesis (*glgC*), fimbriae (*fliF*, *lpfA*, and *fimF*) and SPI-1 genes (*hilA*, *invA*, and *invF*) (Campos-Galvao et al., 2016). *Salmonella* spp. do not encode AHL synthase and do not produce AHL. Instead, *S.* Typhimurium utilizes the SdiA receptor, which is able to sense AHL produced by other bacterial species (Ahmer et al., 1998; Michael et al., 2001; Smith and Ahmer, 2003) when grown on motility agar or during the late exponential phase in liquid culture (Soares and Ahmer, 2011). Environmental conditions such as low pH, high iron concentrations, or growth in spent media, have been shown to repress *sdiA* expression in *S.* Typhimurium. In mice, infection with a *sdiA* mutant lacking AHL signaling resulted in increased fecal shedding and bacterial loads in the livers of infected mice (Volf et al., 2002) indicating that SdiA may be a negative regulator of virulence. Together, these data suggest that while no detectable concentrations of AHL have been found in the mammalian intestine to date, there may be individual bacteria that secrete small quantities of AHL. This idea is supported by the finding that the *Salmonella* AHL-receptor SdiA was activated in the GI tract of turtles colonized with *Aeromonas hydrophila* (Smith et al., 2008). Alternatively, *S.* Typhimurium may utilize the SdiA sensor kinase for other functions such as the detection of other QS molecules such as indole (Lee et al., 2007, 2009), and to facilitate antimicrobial resistance (Nikaido et al., 2012; Vega et al., 2013).

#### AI-2/AI-3

In the mammalian gut two dominating phyla, Bacteroidetes and Firmicutes, are predicted to produce the majority (17 and 83% respectively) of AI-2 (Thompson et al., 2015). Within Firmicutes, 97% of Bacilli, 49% of Clostrida and 27% of other taxonomic classes are predicted to have AI-2 production capabilities (Thompson et al., 2015). Streptomycin has been shown to dramatically shift microbiota composition in the murine gut in the favor of Bacteroidetes (Thompson et al., 2015). However, concurrent introduction of the *E. coli lsrK* mutant, which overproduces AI-2, increased relative abundance of Firmicutes in streptomycin-treated mice and decreased the Bacteroidetes to Firmicutes ratio (Thompson et al., 2015). Thus, AI-2 signaling diminishes the effect of streptomycin-induced dysbiosis on microbiota (Thompson et al., 2015). The microbiota is not the only potential source of AI-2 in the gut. Intriguingly, mammalian cells of epithelial lineage, such as Caco-2 produce a compound which mimics bacterial AI-2 and is detectable by reporter *Vibrio* strain. Increased production of this AI-2 mimic is induced by bacteria, especially when the tight junctions between enterocytes are compromised. This suggests that secretion of signaling molecules, such as the AI-2 mimic by the host allow it to manipulate bacterial behavior in the gut possibly as a protective measure against bacterial transit from the intestinal lumen to the circulatory system (Ismail et al., 2016).

AI-3 signaling has been described in several bacterial species such as *E. coli*, *Salmonella* sp*., Shigella* sp., *Klebsiella pneumoniae*, and *Enterobacter cloacae in vitro* (Sperandio et al., 2003; Walters et al., 2006) and the AI-3 dependent QseC/E system is activated by catecholamines, which are abundant in the mammalian gut (Moreira and Sperandio, 2012). The importance of AI-3 communication in gastrointestinal dysbiosis has not been extensively investigated but might offer unique opportunities for management of gastrointestinal diseases or systemic and behavioral conditions associated with gastrointestinal dysbiosis.

#### Gut Microbiota Is Involved in the Modulation of Catecholamines

The majority of CA [NE, Epi, dopamine (DA)] found in blood and urine is conjugated (glucuronide- or sulfate-conjugated) and biologically inactive (Yoneda et al., 1983; Gaudin et al., 1985). Free CA as well as sulfotransferase enzymes, which conjugate free CA, are gradually distributed in the gut with the lowest concentration in the stomach and highest concentration in the large intestine, which correlates with bacterial loads in these organs (Harris et al., 2000; Asano et al., 2012).

It has been recently discovered that the microbiota are involved in the production of biologically active free CAs in the murine intestine. The majority of the CA in the heavily populated cecum and colon of conventional mice were free (i.e., non-conjugated); while in the sparsely populated ileum a substantial amount of CA was conjugated (Asano et al., 2012). Moreover, the majority of CA in the lumen of germ free (GF) mice was conjugated, and their concentration was lower than in specific pathogen free (SPF) mice. Additionally, β-glucuronidase (GUS) activity, necessary for free CA production, was lower in GF mice. Fecal transplants from SPF mice increased the free CA concentrations and GUS activity in the intestinal lumen of GF mice (Asano et al., 2012). These data indicate the importance of commensal bacteria in GUS-dependent production of biologically active CA in the murine gut.

#### Iron

Iron is essential for the bacterial cell; however, the majority of extracellular iron in the gut is bound by iron-chelating glycoproteins transferrin and lactoferrin. CA can bind to iron within transferrin and lactoferrin, resulting in release of iron from these complexes followed by their uptake by the bacterial siderophores – enterobactin and salmochelin. Porin proteins OmpA and OmpC bring transferrin complexes close to the bacterial cell surface for more efficient uptake of CA-released iron by bacterial siderophores (Freestone et al., 2003; Sandrini et al., 2010, 2013). Porins also allow direct uptake of NE and DA which can enhance bacterial growth (Sandrini et al., 2013). For *Salmonella*, NE-induced growth requires the catecholate siderophore receptors IroN, FepA, and CirA (Williams et al., 2006) as well as siderophore hydrolysis, which liberates siderophore-bound iron in the cytoplasm (Williams et al., 2006; Bearson et al., 2008; Methner et al., 2008). However, the catecholate transport systems may not be required for swine colonization by *S.* Typhimurium (Bearson et al., 2010). The role of QseC/E signaling in NE-dependent bacterial growth needs to be further elucidated (Freestone et al., 2007; Pullinger et al., 2010a).

#### Short Chain Fatty Acids

Microbiota, including pathogenic *E. coli* and *Salmonella* sp., produce short chain fatty acids (SCFAs; acetate, propionate, and butyrate) as the main end-fermentation products that are absorbed by colonic mucosa and used as an energy source by the host and bacteria (Cummings and Macfarlane, 1997). Recent work has demonstrated that microbiota differences between lean and obese children and adolescents correlate with altered proportions of SCFA in plasma and that SCFA are associated with body fat partitioning and *de novo* lipogenesis (Goffredo et al., 2016). In *S.* Typhimurium, SCFAs signal through a two-component regulatory system SirA/BarA primarily at the late exponential phase (Lawhon et al., 2002). BarA is a sensor kinase, which phosphorylates the response regulator SirA in response to extracellular signals. SirA then regulates virulence gene expression through the *csrBC*-CsrA regulatory cascade (Lawhon et al., 2003; Martinez et al., 2011). A mixture of SCFAs similar to that found in the distal ileum was shown to induce the SPI-1 genes *hilA*, *invF*, and *sipC*, while a mixture of SCFAs mimicking the concentration and composition of colonic SCFAs had an opposite effect (Lawhon et al., 2002). Individually, propionate has been shown to decrease SPI-1 expression through the transcriptional regulator HilD (Hung et al., 2013). Furthermore, pre-incubation of *S.* Enteritidis with propionate and butyrate, but not formate, resulted in decreased epithelial cell invasion (Van Immerseel et al., 2003). Finally, in pigs, supplementation with a mixture of organic acids including propionate resulted in decreased *Salmonella* recovery from the mesenteric lymph nodes (Arguello et al., 2013). Thus, dietary supplementation with SCFAs, particularly, propionate, may be a promising intervention strategy for decreasing *Salmonella* loads in farm animals.

#### Indole Secreted by Commensal Bacteria Promotes Intestinal Health and Attenuates *S.* Typhimurium Virulence

To date, over 85 bacterial species have been found to produce the small signaling molecule indole. *E. coli* produces indole during the stationary phase with the help of tryptophanase (TnaA), which converts tryptophan into indole, pyruvate, and ammonia. Indole is generated exclusively by bacteria but can be absorbed by mammalian hosts; therefore, a number of indole derivatives found in blood are entirely dependent on supply of indole from intestinal microbiota (Wikoff et al., 2009). Indole has been shown to promote the health of the gastrointestinal barrier by increasing transepithelial resistance and decreasing inflammatory cytokine (IL-8, IL-10, and NF-κB) secretion and EHEC attachment (Bansal et al., 2010). For *E. coli*, indole has been shown to induce SdiA-dependent and temperature-sensitive reduction in biofilm formation and motility (Lee et al., 2008).

Although *Salmonella* does not produce indole, it is able to sense indole produced by other bacterial species through an unknown mechanism. It has been suggested that indole signaling in *E. coli* occurs via the AHL receptor SdiA. Activation of SdiA by indole leads to reduced biofilm formation as well as reduced acid resistance (Lee et al., 2007, 2009). However, another group determined that an indole associated decrease in biofilm formation in *S.* Typhimurium and *E. coli* was not dependent on SdiA. Instead, high indole concentrations were found to inhibit AHL detection by SdiA (Sabag-Daigle et al., 2012).

Recent studies have shown that *S.* Typhimurium uses the intercellular signaling molecule indole to increase antibiotic tolerance throughout its population by mediating oxidative stress and phage shock response (Vega et al., 2013) as well through induction of multidrug efflux pumps (Nikaido et al., 2008, 2011, 2012). Although indole is the primary metabolite produced from tryptophan by the microbiota, other microbiota-derived tryptophan metabolites exist and likely regulate other microbiota and pathogen properties (Bansal et al., 2007; Lee et al., 2007, 2008).

#### Fucose

Fucose is an abundant sugar in the intestine and its production is microbiota-dependent. For instance, *Bacteroides thetaiotaomicron* facilitates fucose cleavage from glycans such as mucin (Xu et al., 2003). The two-component signal transduction system FusKR of enterohemorrhagic *E. coli* (EHEC) senses fucose and represses LEE expression in the mucus layer, thus preventing energy waste when bacteria are not in close proximity to epithelial cells (Pacheco et al., 2012).

In the intestinal lumen, *S.* Typhimurium expresses fucose utilization proteins FucI, FucU and FucA, which are seldom observed *in vitro* (Ng et al., 2013). It has been proposed that intestinal conditions, such as a shift in gut microbiome population, modulate substrate utilization by the pathogen. *Salmonella* Typhimurium exhibited increased expression of genes involved in metabolism of sialic acid, fucose and propanediol in *B. thetaiotaomicron*-monoassociated mice (Deatherage Kaiser et al., 2013); however, the interplay between fucose sensing and virulence in *S.* Typhimurium is still unknown.

## Gut–Brain Communication in Health and Disease

The bidirectional gut–brain communication involves the central nervous system (CNS), neuroendocrine, neuroimmune, autonomous, and enteric nervous systems (ENS). Neurochemical signals travel from the gut via the afferent neurons while the efferent neurons carry signals from the brain to the gut, connecting the central and peripheral nervous systems. Physiological and psychological stressors induce the release of corticotropin-releasing hormone (CRH) from the hypothalamus which stimulates adrenocorticotropic (ACTH) release by the anterior pituitary gland. ACTH stimulates synthesis and secretion of cortisol by the adrenal cortex. It has been recently recognized that microbiota are involved in the development, function, and communication of the gut–brain, and, in particular, hypothalamic pituitary axis (HPA), which regulates the host response to stress (De Palma et al., 2015; Liang et al., 2015; Frohlich et al., 2016). The catecholamine stress hormone NE is released in the gut via the sympathetic nervous system (Straub et al., 2006). At low and high concentrations NE binds to α and β adrenergic receptors of plasma membranes, respectively (Straub et al., 2006). NE has segment-specific activity in the intestine. Additionally, NE-mediated epithelial response in the proximal and distal colon can be suppressed by α-blocker phentolamine and β-blocker propranolol respectively (Horger et al., 1998). Thus, neuroendocrine-immune intercommunication of the gut–brain axis is important in both the healthy and disease state of animals and humans.

### Gut–Brain Communication: Microbiota to Host

#### Neurochemicals and QS Molecules

The neurotransmitter serotonin [5-hydroxytryptamine (5-HT)], which regulates mood, appetite and sleep is a striking example of a neurochemical which production depends on gut microbiota. The majority (∼90%) of 5-HT is synthesized in the gut (Mawe and Hoffman, 2013) and is microbiota-dependent, because plasma concentrations of serotonin were almost 3-fold higher in the conventional mice compared to GF animals (Wikoff et al., 2009). Hallmark studies determined that gut microbiota, particularly spore-forming bacteria, modulate intestinal metabolites alpha-tocopherol, butyrate, cholate, deoxycholate, *p*-aminobenzoate, propionate, and tyramine to act directly on enterochromaffin cells, stimulating synthesis of serotonin biosynthetic enzyme, Tph1, thus promoting serotonin synthesis (Reigstad et al., 2015; Yano et al., 2015). However, the systemic effect of bacterial regulation of serotonin biosynthesis on distal tissues and organs is unknown (Mawe and Hoffman, 2013).

Tryptophan is another example of microbiota-dependent metabolism of signaling molecules. Serum tryptophan concentrations were significantly higher in GF mice since they were lacking microbes that convert tryptophan to indole. Additionally, an indole metabolite, indole-3-propionic acid (IPA), has been identified only in plasma of conventional mice and colonization of GF mice with the commensal bacterium, *C. sporogenes*, restored IPA in the serum of GF mice to the levels found in conventional mice (Wikoff et al., 2009).

Sex hormones have also been implicated in the modulation of the brain-gut axis at the CNS, autonomous NS and ENS levels with certain differences seen between males and females in pain perception and HPA response. Brain-microbiota communication may also be modulated by sex hormones (Mulak et al., 2014), while bacteria can synthesize steroid metabolizing enzymes thus modulating the host (Bokkenheuser and Winter, 1980). For example, microbial β-glucuronidase deconjugates estrogens in the gut and makes them available for reabsorption via enterohepatic circulation (Flores et al., 2012; Markle et al., 2013). Additionally, *S*. Typhimurium has been found to alter multiple host metabolic pathways, particularly those that regulate synthesis and degradation of steroid hormones (Antunes et al., 2011). Finally, the microbiota is required for biologically active CA production (NE, Epi, and dopamine) in the intestinal lumen, which can be utilized by the host and microbiota (Asano et al., 2012). Conversely, estrogen receptor β (ERβ) status affects species richness and relative abundance of prominent phylotypes of murine fecal microbiota composition, where relative abundance of Bacteroidetes and Proteobacteria was higher in ERβ^+/+^ ERβ^-/-^ and mice (Menon et al., 2013).

#### Microbiota Regulates the Hypothalamic Pituitary Axis

It is known that microbiota aids in the brain development and metabolism (reviewed in Goyal et al., 2015). Additionally, gut microbiota plays an important role in regulation of the HPA, particularly, in modulating the host’s response to stress (Dinan and Cryan, 2012). For example, the gut microbiota initiates signaling which affects neurons involved in motor control and anxiety-like behavior (Diaz Heijtz et al., 2011) independently of inflammation, GI, or vagal signaling (Bercik et al., 2011). In another study, GF rats exhibited decreased social communication, increased anxiety-like behavior and neuroendocrine response to acute stress (Crumeyrolle-Arias et al., 2014). Finally, GF mice had exaggerated responses to stress indicated by the elevated concentration of stress hormones ACTH and corticosterone relative to their non-GF genetically similar counterparts. GF mice also expressed decreased levels of brain-derived neurotrophic factor in the cortex and hippocampus, which was reversed by introducing *Bifidobacterium infantis* in the early postnatal stage (Sudo et al., 2004). Together these studies demonstrate the importance of microbiota in the host response to stress mediated by the HPA axis.

There is further evidence that hyper-responsiveness of the HPA can be reversed by probiotic bacterial species. In mice, supplementation with *Lactobacillus rhamnosus* (*JB*-1) has been shown to regulate the expression of the main CNS inhibitory neurotransmitter γ-aminobutyric acid (GABA) (Bravo et al., 2011). Furthermore, supplementation with *L. rhamnosus* decreased plasma concentrations of corticosterone and resulted in reduced anxiety and depression-like behavior in mice (Bravo et al., 2011). Another probiotic, *Bifidobacterium longum* NCC3001, normalized anxiety-like behavior when given to mice with chronic colitis (Bercik et al., 2011). Additionally, administration of a probiotic formulation consisting of *Lactobacillus helveticus* R0052 and *B. longum* R0175 for 30 days decreased anxiety-like behavior in rats and alleviated physiological distress and urinary cortisol in healthy humans (Messaoudi et al., 2011).

It has been determined that exposure to stressors, including social stress and response to environmental stressors, changes composition and density of the gut microbial community (Bailey and Coe, 1999). For instance, social stress decreased the genus *Bacteroides* and increased the abundance of the genus *Clostridium* in mice. Stress induced an increase of serum concentrations of cytokines IL-6 and MCP-1, which were inversely correlated with the relative abundance of *Coprococcus*, *Dorea*, and *Pseudobutyrivibrio* species (Bailey et al., 2011). Interestingly, mice pre-treated with antimicrobials to reduce microbiota did not develop increases in IL-6 and MCP-1, suggesting that the observed increase in cytokines in stressed mice was induced by microbiota (Bailey et al., 2011). Research has shown that stress in early life, such as maternal separation, alters behavior, immunity and microbiota in rats. Stressed pups exhibited increased systemic concentrations of plasma corticosterone as well as TNF-α and INF-γ along with increased visceral sensation (Gareau et al., 2007; O’Mahony et al., 2009). Administration of probiotics ameliorated the signs of gut dysfunction and decreased corticosterone concentrations in these rat pups (Gareau et al., 2007). The probiotic *Lactobacillus farciminis* was found to be effective in alleviating intestinal permeability as well as corticosterone, ACTH and pro-inflammatory cytokine concentrations induced by LPS treatment in female rats (Ait-Belgnaoui et al., 2012).

Temperament in animals is associated with responsiveness of the HPA and resting levels of cortisol (Curley et al., 2008; Schuehle Pfeiffer et al., 2009) and differential immune response to bacterial infection (Burdick et al., 2011). In mice, gut microbiota may regulate the HPA and stress responsiveness, possibly by directly regulating neuronal expansion and morphology. For example, microbiota-deficient mice exhibited expansion of amygdala and hippocampus (Luczynski et al., 2016). Interestingly, in young children an association has been discovered between temperament and the microbiota (Christian et al., 2015). Particularly, increased surgency/extraversion, sociability and high-intensity pleasure was associated with increased alpha diversity in both sexes (Christian et al., 2015). In finishing cattle calm temperament was associated with increased *E. coli* O157:H7 shedding following transportation stress (Schuehle Pfeiffer et al., 2009).

### Stress and Microbiota: Role in *S.* Typhimurium Pathogenesis

*Salmonella* Typhimurium induces enteritis characterized by neutrophil infiltration, submucosal edema, goblet cell depletion, epithelial disruption, and crypt abscesses (Fabrega and Vila, 2013). In the gut, *Salmonella* employs strategies that allow it to compete with resident microbiota and eventually overcome colonization resistance. Ultimately, *Salmonella*-induced disruption of the microbiota may disrupt regulation of the nervous system by microbiota-derived metabolites.

#### Dysbiosis Benefits *S.* Typhimurium

Disturbance in microbiota density and composition have long been associated with increased susceptibility to salmonellosis. Mice infected with *S.* Typhimurium suffer systemic infection but about 95% do not develop gastrointestinal illness characteristic of *S.* Typhimurium infection in humans. Treatment of mice with a single dose of streptomycin 24 h prior to intragastric inoculation with *S.* Typhimurium leads to dramatically reduced microbiota density and diversity (Barthel et al., 2003). Consequently, transient disruption of colonization resistance allows *S.* Typhimurium to establish itself in the cecum and colon as fast as 8–12 h post-infection, and to induce acute mucosal inflammation in mice. These observations have led to use of the streptomycin-treated mouse as a model of *Salmonella-*induced diarrhea in humans (Croswell et al., 2009; Kaiser et al., 2012). There is a strong correlation between an antibiotic-induced (streptomycin, vancomycin, and metronidazole) microbiota shift and subsequent severity of *S.* Typhimurium-induced infection (Ferreira et al., 2011). Antibiotic treatment leads to a steep decrease in total bacterial microbiota numbers and is associated with increased systemic translocation of *S.* Typhimurium and prolonged intestinal inflammation relative to untreated mice infected with *S.* Typhimurium (Croswell et al., 2009).

**FIGURE 4:**
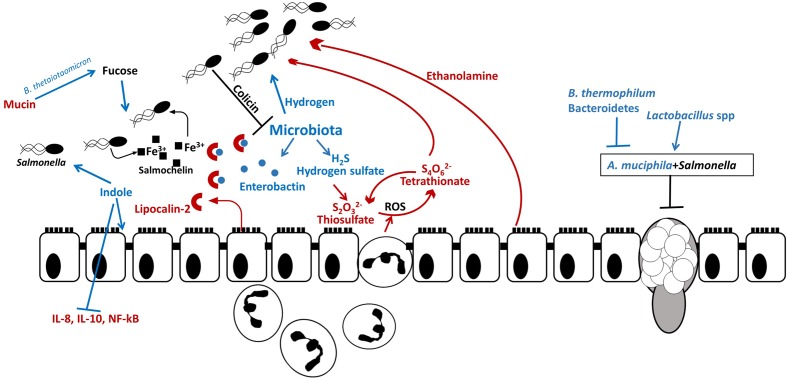
***Salmonella* outcompetes resident microbiota in the gut.** Host and resident microbiota protects against *Salmonella* colonization: indole secreted by commensals promotes intestinal health by decreasing secretion of proinflammatory cytokines and by decreasing *Salmonella* virulence gene expression and certain bacteria (*B. thermophilum* and Bacteroidetes) are associated with increased resistance to *Salmonella. Salmonella* uses microbiota-produced hydrogen for initial establishment in the intestine. *Salmonella* induces secretion of ROS and antimicrobial peptides by neutrophils and epithelial cells. While *Salmonella* is resistant to ROS, they are harmful to commensal microbes. In the inflamed intestine, microbiota-produced hydrogen sulfate is converted by the host to tetrathionate, which is used by *Salmonella* as an electron acceptor for anaerobic respiration and allows *Salmonella* to utilize ethanolamine, produced by the host as carbon source. Additionally, *Salmonella* – produced bacteriocin, colicin, inhibits resident *Escherichia coli* in the intestine. Thus *Salmonella*-induced inflammation leads to dysbiosis and allows *Salmonella* to propagate in the gut. Some resident bacterial species contribute to *Salmonella* infection. For example, *A. muciphila* inhibits mucin production and aids *Salmonella* in decreasing goblet cell population. Additionally, fucose, which is cleaved from mucin by *B. thetaiotaomicron* is utilized by *Salmonella* at the initial colonization stage.

Intestinal inflammation helps WT and avirulent *Salmonella* to compete with microbiota and enhances *Salmonella* colonization in mice (**Figure 4**). However, the avirulent strain, which lacked two virulence-associated type III secretion systems, but not WT *Salmonella* was outcompeted by microbiota in the streptomycin mouse model (Stecher et al., 2007). Additionally, WT was able to alter the microbiota composition (*Salmonella* was the predominant species, ∼90%) in streptomycin-treated mice compared to an avirulent strain (Stecher et al., 2007).

#### *Salmonella* Typhimurium Induces Dysbiosis

*Salmonella* Typhimurium is able to modify gut microbiota even without antimicrobial pre-treatment. For example, oral *S.* Typhimurium infection induces SPI-1-dependent dysbiosis which is reversed within a month after inoculation (Barman et al., 2008). Interestingly, a change in microbiota composition preceded *S.* Typhimurium-induced diarrhea, suggesting a host-pathogen interaction resulting in dysbiosis but unrelated to diarrhea (Barman et al., 2008). One of the drivers of *Salmonella*-induced dysbiosis is the depletion of Clostridia, which are the main butyrate producers. Decrease of cecal butyrate leads to elevated oxygenation of colonocytes and increased aerobic proliferation of Bacilli, Bacteroidia, and Gammaproteobacteria (including *S.* Typhimurium) (Rivera-Chavez et al., 2016). Additionally, mice with low complexity microbiota (8–40 bacterial strains) were susceptible to *S.* Typhimurium-induced colitis even in the absence of pretreatment with streptomycin. Clearance of *Salmonella* can be facilitated by transferring microbiota from conventional mice to mice with the low complexity microbiota (Endt et al., 2010; Stecher et al., 2010). Interestingly, mice colonized with commensal *E. coli* were more susceptible to *Salmonella*-induced inflammation, suggesting that closely related phylotypes displayed correlated abundances and pathogen blooms (Stecher et al., 2010). These differences are not limited to mammalian hosts. In newly hatched chicks, *S.* Typhimurium does not cause clinical disease; however, it induces minor changes in the cecal microbiota (Juricova et al., 2013). In adult chickens, *S.* Typhimurium infection led to an increase in *Enterobacteriaceae* and decrease in *Ruminococcaceae* in the cecum (Videnska et al., 2013) although, overall changes in microbiota composition were much less pronounced compared to mammals.

Microbial metabolites can be beneficial for the initial pathogen assimilation in the gut. It has been shown that murine cecal microbiota produce approximately 2 μM hydrogen (Maier et al., 2013). Moreover, the genes encoding enzymes involved in the hydrogen production are abundant within the gut microbiota. In *S.* Typhimurium, hydrogen-consuming hydrogenases (*hyd*) are required for initial establishment in the intestine (Maier et al., 2004, 2013, 2014) and overcoming colonization resistance in SPF mice, whereas in the GF mouse the ability to consume hydrogen is not required (Maier et al., 2013). Interestingly, the inability of a *hyd3* mutant to consume microbiota-derived hydrogen did not affect *S.* Typhimurium colonization of systemic sites in the mouse (Maier et al., 2014). Therefore, hydrogen utilization is considered to be a general feature of *S.* Typhimurium necessary for gut colonization.

#### *Salmonella* Typhimurium Exploits Intestinal Inflammation to Compete with Microbiota

Intestinal inflammation provides metabolic advantages to *Salmonella* to compete with resident microorganisms. Under normal conditions, the mammalian gut converts harmful hydrogen sulfide to thiosulfate (Furne et al., 2001). During inflammation induced by *S.* Typhimurium virulence factors SPI-1 and SPI-2, thiosulfate is oxidized to tetrathionate by reactive oxygen species (ROS). Subsequently, *S.* Typhimurium utilizes tetrathionate as an electron acceptor for anaerobic respiration, which allows the pathogen to outcompete the microbiota in the inflamed gut (Winter et al., 2010). Tetrathionate also acts as an electron acceptor allowing *S.* Typhimurium to utilize ethanolamine released from the host tissue. The *ttrA* gene encoding tetrathionate reductase subunit A is essential for *S.* Typhimurium growth in ethanolamine *in vivo* while the majority of gut microbiota are unable to use ethanolamine for fermentation (Thiennimitr et al., 2011).

**FIGURE 5:**
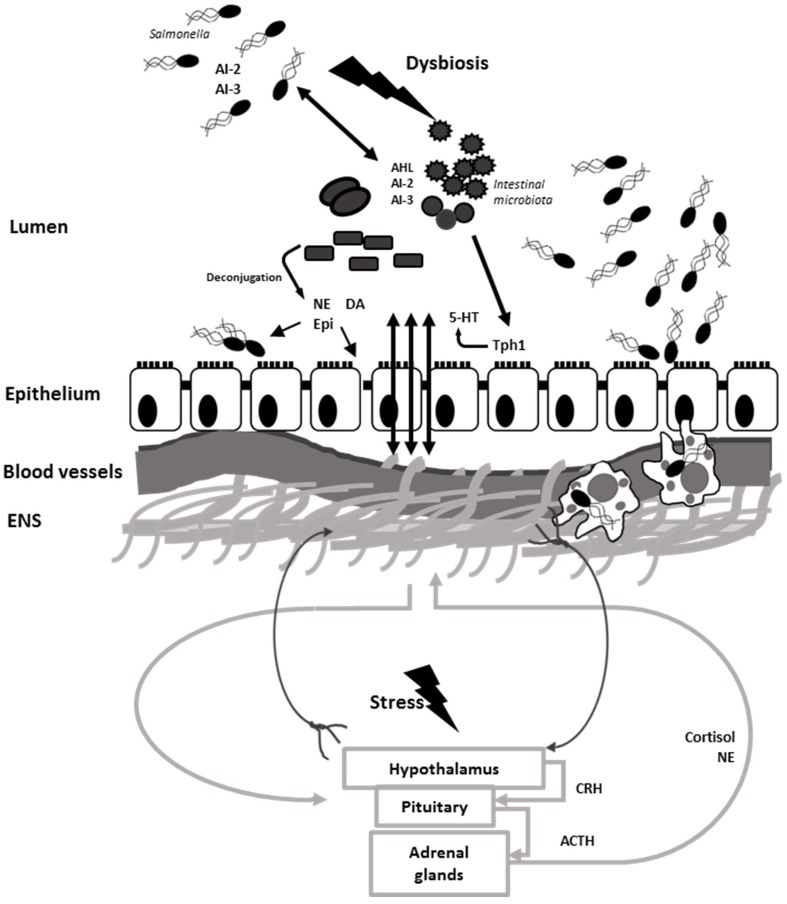
***Salmonella* induces dysbiosis and interferes with gut–brain communication.** Neural signals travel via efferent neurons from CNS to the ENS and back via afferent neurons. Microbiota stimulate synthesis and modulates bioavailability of neurochemicals such as 5-HT, NE, DA, and Epi which are utilized by the host. Stress stimulates the activation of HPA axis and release of cortisol and catecholamines. NE is released in the gut via spillover from sympathetic neurons. Resident microbiota use an array of quorum sensing signals which can be intercept by *Salmonella*. Catecholamines along with AI-2 and AI-3 QS molecules stimulate *Salmonella* growth and virulence in the intestine. Stress aids to *Salmonella* pathogenesis and induces dysbiosis, which helps *Salmonella* establishment in the intestine and dissemination via phagocytic cells.

*Salmonella* Typhimurium employs efficient mechanisms to compete for nutritional iron in the inflamed intestine. One of the mechanisms the pathogen uses is an induction of lipocalin-2 production by neutrophils. Lipocalin-2 is an antimicrobial protein which prevents iron acquisition in the intestine by commensal bacteria by binding to the siderophore, enterobactin. *Salmonella* Typhimurium produces a glycosylated variant of enterobactin, salmochelin, which utilizes transporter IroN. Salmochelin is not bound by lipocalin-2 and, therefore, gives *Salmonella* an advantage over IroN-negative bacteria (Raffatellu et al., 2009). The lipocalin-2 resistance system has also been found in non-pathogenic Nissle strain of *E. coli* which is able to outcompete *S.* Typhimurium in a mixed infection and reduce pathogen numbers, making it a promising probiotic for preventative and therapeutic use (Deriu et al., 2013). *Salmonella* Typhimurium also utilizes ferrous iron as co-repressor of siderophore synthesis in order to avoid damage by ROS secreted by immune cells. Production of iron-depleting agents such as lipocalin-2, lactoferrin, ROS and RNS by macrophages triggers ferric uptake regulator (*fur*)-dependent responses which control defense against peroxide. In addition to defensive mechanisms *S.* Typhimurium employs offensive tactics to compete in the gut such as production of antimicrobial bacteriocins. Intestinal inflammation triggers pore-forming, *fur*-dependent bacteriocin, colicin lb, which gives *Salmonella* an advantage over colicin-sensitive *E. coli* strains and allows *Salmonella* to “bloom” in the inflamed gut (Nedialkova et al., 2014). In addition to inducing intestinal blooms of the pathogen, intestinal inflammation combined with microbiota disturbance has been shown to increase genetic material exchange between *Salmonella* and *E. coli* which could be prevented by commensal microbiota (Stecher et al., 2012). Disruption of the microbiota leads to disruption of the communication between the microbiota and brain through altered chemical signaling between microbiota, gut, and brain (**Figure 5**).

#### Microbiota Regulates Susceptibility to Infection

Some commensal bacteria develop synergistic relationships with *S.* Typhimurium. One such example is *Akkermansia muciphila* which causes increased pathology during *S.* Typhimurium infection by disturbing mucus production and sulfation. A combination of *A. muciphila* and *S.* Typhimurium significantly reduced numbers of mucin-filled goblet cells in the murine intestine, compared to uninfected mice or mice inoculated with either *A. muciphila* or *S.* Typhimurium alone. Presence of both bacteria increased *S.* Typhimurium proportions to 94% from 2.2% in an *S.* Typhimurium mono-infection. Additionally, expression levels of inflammatory cytokines INF-γ, IP-10, TNF-α, IL-12, IL-6, and IL-17 were significantly increased in mice infected with both bacteria compared to a single infection or uninfected mice. Thus, during *S.* Typhimurium infection commensal *A. muciphila* turns into a pathobiont and contributes to disease symptoms (Ganesh et al., 2013).

The gut microbiota induces systemic IgG production in response to GI infection, protecting the host from *Salmonella*-induced bacteremia. IgG production is accomplished in large part through TLR4 signaling in response to gram-negative murein lipoprotein (Zeng et al., 2016). Particular members of the resident microbial community have been associated with increased resistance to infection. A study performed in conventional streptomycin-treated mice has shown that the Bacteroidetes phylum was associated with increased resistance to *S.* Typhimurium-induced colitis while *Lactobacillus* sp. was associated with decreased resistance (Ferreira et al., 2011). Also, a combination of commensal microbiota, particularly *Clostridium citroniae*, and gut metabolites exhibited antibacterial activity toward *S.* Typhimurium as well as reduced cell invasion (Antunes et al., 2014). Finally, supplementation with the probiotic *Bifidobacterium thermophilum* RBL67 in combination with prebiotics [fructo- (FOS), galacto- (GOS), and mannan- (MOS) oligosaccharides] decreased *S.* Typhimurium colonization in a swine proximal colon simulation model (Tanner et al., 2014). Co-culture of *B. thermophilum* RBL67 and *S.* Typhimurium at mid-exponential growth phase resulted in repression of regulatory gene *phoP* and flagella, although expression of SPI-1 genes, SPI-2 genes, and fimbriae was increased. The premature induction of virulence gene expression and reduced motility in *S*. Typhimurium induced by *B. thermophilum* RBL67 resulted in restricted colonization by the pathogen (Tanner et al., 2016).

### Stress Can Modify the Interaction between Host, Microbiota and *Salmonella*

Exposure to stress hormones has been shown to increase survival and virulence of *Salmonella* (by the mechanisms described above). For example, exposure of *S.* Typhi to NE and Epi induced release of toxin hemolysin E, which was attenuated by the β blocker propranolol (Karavolos et al., 2011). In another study treatment of intestinal tissues with 10 μM NE improved the uptake of *S.* Choleraesuis into porcine jejunal Peyer’s patches (Green et al., 2003). Likewise, incubation of *S.* Typhimurium in serum-SAPI medium with 2 mM NE prior to inoculation increased bacterial survival in the swine stomach and bacterial loads in the small intestine, large intestine and feces compared to bacteria grown in LB without NE (Toscano et al., 2007). It was determined that density of the bacterial inoculum should be low for efficient NE-induced bacterial growth (O’Donnell et al., 2006). Additionally, CAs can alter cellular uptake of *Salmonella* at early stages of infection. For example, increased dopamine concentrations at the contra-luminal aspect of the intestine resulted in decreased bacterial recovery from Payer’s patches *ex vivo* (Brown and Price, 2008) whereas cortisol increased intracellular *S.* Typhimurium proliferation in primary porcine alveolar macrophage cultures and in mice (Verbrugghe et al., 2011). The above mentioned effect is attributed to the cortisol-induced activation of regulator protein ScsA in *S.* Typhimurium which leads to increased SCV-production by macrophages allowing increased bacterial proliferation (Verbrugghe et al., 2016).

Not only does stress in the host aid the virulence of the pathogen, but it also increases host susceptibility to *S.* Typhimurium infection. To illustrate, feed withdrawal and heat stress in chickens were associated with increased *S.* Typhimurium numbers and increased intestinal pathology (Burkholder et al., 2008). Treatment of pigs with NE or the NE precursor, 6-hydroxydopamine (6-OHDA), which mimics acute stress, increased *Salmonella* shedding in pigs (Pullinger et al., 2010b). Additionally, decreased fecal shedding exhibited by the *qseC* mutant was reversed by treatment of pigs with 6-OHDA prior to infection (Pullinger et al., 2010b) which suggests that activation of QseC-dependent signaling by NE may not be required for *S.* Typhimurium virulence *in vivo*, as long there is an increase in stress hormone levels in the host. Another study demonstrated that, 24 h feed withdrawal or dexamethasone injections increased serum cortisol concentrations and *Salmonella* shedding in pigs (Verbrugghe et al., 2011).

Finally, a combination of stress and bacterial infection can affect resident microbiota in the GI tract (Tannock and Savage, 1974) as well as gut–brain communication. For example, acute stress combined with *C. rodentium* infection induced memory dysfunction in mice, while infection alone did not result in memory impairment. Pigs infected with *Salmonella* exhibited subtle behavioral change as compared to uninfected control pigs despite having no obvious clinical signs of infection (Rostagno et al., 2011). Daily treatment with probiotics (*L. rhamnosus* R0011 and *L. helveticus* R0052) restored microbiota and prevented memory dysfunction in infected stressed mice. Probiotics also decreased serum corticosterone in mice and pro-inflammatory cytokine concentrations in these animals indicating that beneficial bacteria may ameliorate gastrointestinal illness and prevent memory alteration (Gareau et al., 2011). It is unclear whether the exposure to stress during infection with gastrointestinal pathogens such as *S*. Typhimurium will affect memory or other brain functions in animals and additional study is warranted.

## Concluding Remarks and Future Perspectives

The evidence supports the hypothesis that microbiota communicates with the CNS and ENS through neural, endocrine, immune and humoral links (Carabotti et al., 2015; Brown and Clarke, 2016) resulting in the microbiota having numerous effects on the health of the host (Robles Alonso and Guarner, 2013). The gut microbiota helps shape and is shaped by the innate and adaptive immune systems and plays a role in host nutrition and neuroendocrine pathways (Galland, 2014; Marietta et al., 2015; Tomkovich and Jobin, 2016). Further, mounting evidence suggests that the gastrointestinal microbiota is involved in the development and function of the mammalian nervous system (Neufeld et al., 2011; Jasarevic et al., 2015).

The enteric pathogen *S*. Typhimurium employs a multitude of tactics to overcome colonization resistance by microbiota including (1) inducing inflammation which modulates nutrient availability by suppressing other bacteria; (2) through complex gene regulation pathways that link quorum signaling and metabolism; and (3) intercepting signals produced by the microbiota and host.

There is relatively little information on the effects of neuroendocrine hormones on *Salmonella* gene expression and virulence in animals despite decades of work suggesting that stress is associated with *Salmonella* shedding from people and animals. Future research should further investigate the multidirectional signaling in the GI tract to aid in our understanding of the communication between the host, resident microbiota and GI pathogens within the challenging environment of gastrointestinal tract.

## Author Contributions

All authors listed, have made substantial, direct and influential contribution to the work and approved it for publication.

## Conflict of Interest Statement

The authors declare that the research was conducted in the absence of any commercial or financial relationships that could be construed as a potential conflict of interest.
